# Limitation of treatment in prehospital care – the experiences of helicopter emergency medical service physicians in a nationwide multicentre survey

**DOI:** 10.1186/s13049-019-0663-x

**Published:** 2019-10-02

**Authors:** Heidi Kangasniemi, Piritta Setälä, Heini Huhtala, Antti Kämäräinen, Ilkka Virkkunen, Joonas Tirkkonen, Arvi Yli-Hankala, Sanna Hoppu

**Affiliations:** 1 Research and Development Unit, FinnHEMS Ltd, WTC Helsinki Airport, Lentäjäntie 3, 01530 Vantaa, Finland; 2 Division of Anaesthesiology, Department of Perioperative, Intensive Care and Pain Medicine, University of Helsinki and Helsinki University Hospital, Töölö Hospital, Topeliuksenkatu 5, FIN-00029 HUS, Helsinki, Finland; 3 Faculty of Medicine and Life Sciences, Tampere University, FI-33014 Tampere, Finland; 4 Emergency Medical Services, Tampere University Hospital, P.O. Box 2000, FI-33521 Tampere, Finland; 5 Faculty of Social Sciences, Tampere University, P.O. Box 100, FI-33014 Tampere, Finland; 6 Department of Anaesthesia, Tampere University Hospital, P.O. Box 2000, FI-33521 Tampere, Finland

**Keywords:** Emergency medical services, Treatment limitations, Ethics, Nursing home, DNAR, Decision-making

## Abstract

**Background:**

Making ethically sound treatment limitations in prehospital care is a complex topic. Helicopter Emergency Medical Service (HEMS) physicians were surveyed on their experiences with limitations of care orders in the prehospital setting, including situations where they are dispatched to healthcare facilities or nursing homes.

**Methods:**

A nationwide multicentre study was conducted among all HEMS physicians in Finland in 2017 using a questionnaire with closed five-point Likert-scale questions and open questions. The Ethics Committee of the Tampere University Hospital approved the study protocol (R15048).

**Results:**

Fifty-nine (88%) physicians responded. Their median age was 43 (IQR 38–47) and median medical working experience was 15 (IQR 10–20) years. All respondents made limitation of care orders and 39% made them often. Three fourths (75%) of the physicians were often dispatched to healthcare facilities and nursing homes and the majority (93%) regularly met patients who should have already had a valid limitation of care order. Every other physician (49%) had sometimes decided not to implement a medically justifiable limitation of care order because they wanted to avoid conflicts with the patient and/or the next of kin and/or other healthcare staff. Limitation of care order practices varied between the respondents, but neither age nor working experience explained these differences in answers. Most physicians (85%) stated that limitations of care orders are part of their work and 81% did not find them especially burdensome. The most challenging patient groups for treatment limitations were the under-aged patients, the severely disabled patients and the patients in healthcare facilities or residing in nursing homes.

**Conclusion:**

Making limitation of care orders is an important but often invisible part of a HEMS physician’s work. HEMS physicians expressed that patients in long-term care were often without limitations of care orders in situations where an order would have been ethically in accordance with the patient’s best interests.

**Electronic supplementary material:**

The online version of this article (10.1186/s13049-019-0663-x) contains supplementary material, which is available to authorized users.

## Introduction

Physician-staffed helicopter emergency medical service (HEMS) generally represents the highest level of care available in the prehospital setting. HEMS physicians have advanced experience in initiating a vast array of life-sustaining therapies at the site of the patient [1, 2]. HEMS units are dispatched to all high-risk medical situations based on the provision of medical equality in Finland, therefore they are also dispatched to healthcare facilities (HCFs) and nursing homes (NHs) [3]. However, an acute critical illness may be a manifestation of the terminal phase of the chronic condition rather than an unpredictable event among the patients in HCFs and NHs [4, 5]. Often the same factors that have led to the need for 24-h care and dependence in activities of daily living may lead to the withholding or withdrawing of life-sustaining therapies in acute situations [6].

There are numerous reports on ethical issues concerning withholding cardiopulmonary resuscitation (CPR) [7–10], but studies on limitation of care orders (LCO) beyond ‘do not attempt resuscitation’ (DNAR) are scarce, especially in the prehospital setting [11–13]. As far as we know, there are only a few studies on prehospital providers’ end-of-life decision-making in HCFs and NHs [14, 15]. Here we describe the HEMS physicians’ experiences with the LCOs they make in HCFs, NHs, and prehospital settings.

## Methods

### Design and ethics

We conducted a cross-sectional nationwide multicentre study among all HEMS physicians in Finland between 20th January and 30th April 2017. We designed the study survey around ethical dilemmas described in the recent literature [16–19]. An independent senior physician evaluated the feasibility of the questionnaire and appropriate revisions were conducted. The Ethics Committee of the Tampere University Hospital approved the study protocol (Approval no: R15048). The study was accepted by all Finnish university hospitals, the National Institute for Health and Welfare, and FinnHEMS Ltd. Participation was voluntary. We informed the physicians about the study with a personal or recorded video presentation and written information. The existing LCO guidelines weren’t presented while giving this information. Absent respondents were contacted via email with a printable version of the questionnaire.

### Setting

The Finnish emergency medical service (EMS) system has been previously described in detail [20]. In short, a HEMS unit is dispatched to all severely ill or injured patients alongside an advanced life support (ALS) unit by a national emergency dispatch center. The HEMS crew consists of a HEMS physician, a pilot, and a HEMS nurse-paramedic. HEMS services are coordinated by FinnHEMS Ltd., which is a publicly financed, non-profit corporation jointly owned by all Finnish university hospital districts. HEMS bases operate 24 h a day. There are six HEMS bases of which five are physician-staffed and one is HEMS-paramedic-staffed. Most HEMS physicians are specialists in anaesthesiology and intensive care medicine.

The Finnish healthcare system with HCFs, NHs, and care for the aged in general has also been described in the literature [21, 22]. In this study, the term ‘HCF’ included municipal health centers, hospitals, and private clinics. The term ‘NH’ refers to all the various housing services, which include residential homes for older people, sheltered housing with and without 24-h assistance, institutions for people with intellectual disabilities, institutions for substance abusers, rehabilitation institutes, and hospice units. Many NHs are private, whereas most of the HCFs providing institutional long-term care are public. Both HCFs and NHs usually have skilled healthcare staff, and both can utilise the public EMS system for the treatment and/or transportation of patients in acute situations. All patients with chronic illnesses should have a treatment plan according to the national guidance [23, 24]. If a patient is admitted to 24-h care, the attending physician should draft an emergency care plan and/or an anticipatory end-of-life care plan [3, 24–26].

### Limitation of care orders

Finnish legislation emphasises that the patient’s wishes should always be respected when planning his/her treatment and when this is not possible, the plan should represent the patient’s assumed best interests [23, 27]. A senior physician may limit any medical treatment considered futile, and the patient has the right to refuse any treatment offered. Ineffective or harmful therapies may not be provided even if they are demanded by the patient or relatives. The patient can create an advance directive (AD) to limit his/her treatment. All LCOs and ADs should be clearly stated in the patient’s medical records. The most common AD/LCO is DNAR. Other limitations usually concern intensive care, intubation, mechanical ventilation, invasive procedures, and intravenous antibiotics, transferring the patient to a hospital, and feeding or hydrating the patient intravenously or enterally. Palliative care and terminal care are often accompanied by DNAR and the limitation of intensive care, but these preferences need to be stated separately.

In the prehospital setting, paramedics can independently withhold a cardiopulmonary resuscitation attempt if there are secondary signs of death, obviously lethal trauma, or an existing DNAR order [8]. Paramedics can withdraw a resuscitation attempt after consulting the HEMS physician in cases of unwitnessed cardiac arrest, prolonged downtime, or end-stage chronic medical conditions [8, 20]. The HEMS physician can make a LCO via phone if needed and may cancel the HEMS unit’s participation in certain missions if he/she assesses that adequate medical resources are already at the site of the patient or after making a LCO.

### Measures and statistics

We collected demographic data on the physicians’ HEMS unit, age, gender, specialty and all previous work experience within the medical field. Our survey with 38 questions explored their opinions, attitudes, and experiences with prehospital LCOs in general, HEMS missions designated to HCF and NHs, and the LCOs set in those places. The closed questions or claims were answered with five-point Likert-scale choices with the sixth response choice being ‘I wish not to answer this question’. The open questions addressed the features and challenges of prehospital LCOs. The questionnaire was given in Finnish, and the English translation is provided in Additional file 1.

Statistical analyses were performed using SPSS applications (IBM SPSS Statistics for Macintosh, Version 24.0, Armonk, NY: IBM Corp). We described the material with descriptive statistics (measures of central tendency and spread, and graphs) and differences based on the demographics of the physicians. We compared the answers in the following demographic groups: men and women, the age of the physician, and work experience in years. We analysed the Likert-scale answers with contingency tables, the Chi-Square or Fisher’s exact test, and a Spearman correlation [28]. A *p*-value < 0.05 was considered statistically significant, and all tests were two-sided. For the qualitative data, we used content analysis to evaluate the information from the material and quantified the most commonly occurring reduced expressions [29].

## Results

The total number of HEMS physicians during the study period was 67, and they were equally distributed to the five helicopter bases. Fifty-nine (88%) HEMS physicians participated in the study and the response rates by bases varied between 69 and 100%. The respondents were mainly experienced anaesthesiologists (Table 1).

**Table 1:** Sociodemographic data of Helicopter Emergency Medical Service (HEMS) physicians in Finland in 2017

Sociodemographic background of the respondents *n* = 59	*n*	%
Gender		
Men	39	66
Women	20	34
Age		
Mean, years (SD)	43	(6.02)
Min – max, years	31–59	
First specialty		
Anaesthesiology and Intensive Care	53	90
Internal Medicine	3	5
Emergency Medicine	2	3
General Medicine	1	2
Specialization status		
Specialized	52	91
Specializing	5	9
Not responded	2	3
Second specialty; Emergency Medicine	9	15
Specialized	5	8
Specializing	4	7
Special competence (SC)^a^	37	63
1 SC, Emergency medical services (EMS)	26	44
1 SC, other than EMS	4	7
2 SC, EMS and some other	5	8
2 SC, both other than EMS	1	2
3 SC, EMS and two other SCs	1	2
Work experience in EMS		
Median, years (Q1–Q3)	10	(6–16)
Min – max, years	1–27	
Work experience as physician		
Median, years (Q1–Q3)	15	(10–20)
Min – max, years	1^b^ – 33	
Work experience as EMS physician (*n* = 55)		
Median, years (Q1–Q3)	8.5	(5–13)
Min – max, years	0.5^b^ – 24	

^a^ The Finnish Medical Association can bestow special competences as additional to the official specialisation system. Special competences relate to certain specialty areas that particular skills are demanded (https://www.laakariliitto.fi/koulutus/erityispatevyydet/ohjeet/)

^b^ One experienced HEMS paramedic had recently graduated from medical school

### HEMS physicians’ attitudes, opinions, and experiences with LCOs

**Fig. 1:**
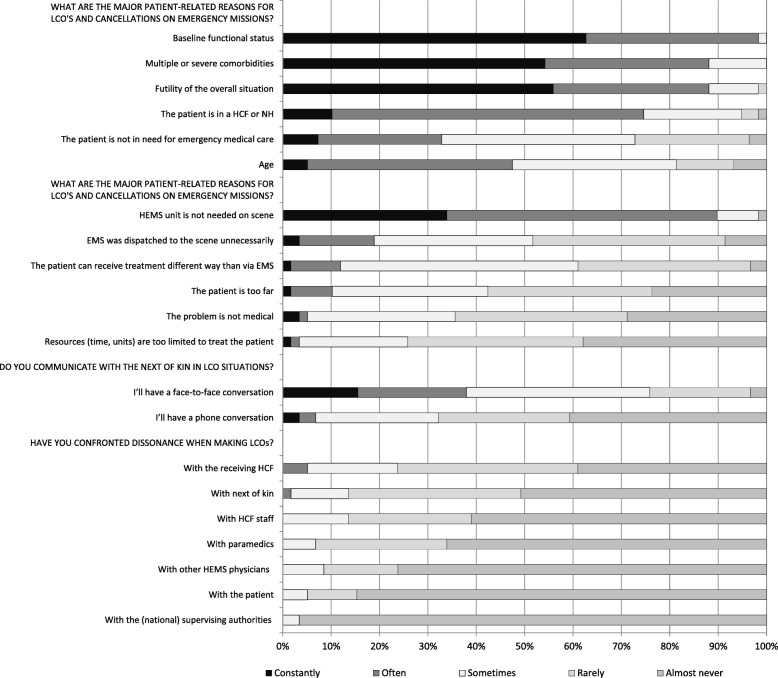
The practices of Finnish HEMS physicians (*n* = 59) to make limitation of care orders (LCO). HCF is a healthcare facility and NH is a nursing home

**Fig. 2:**
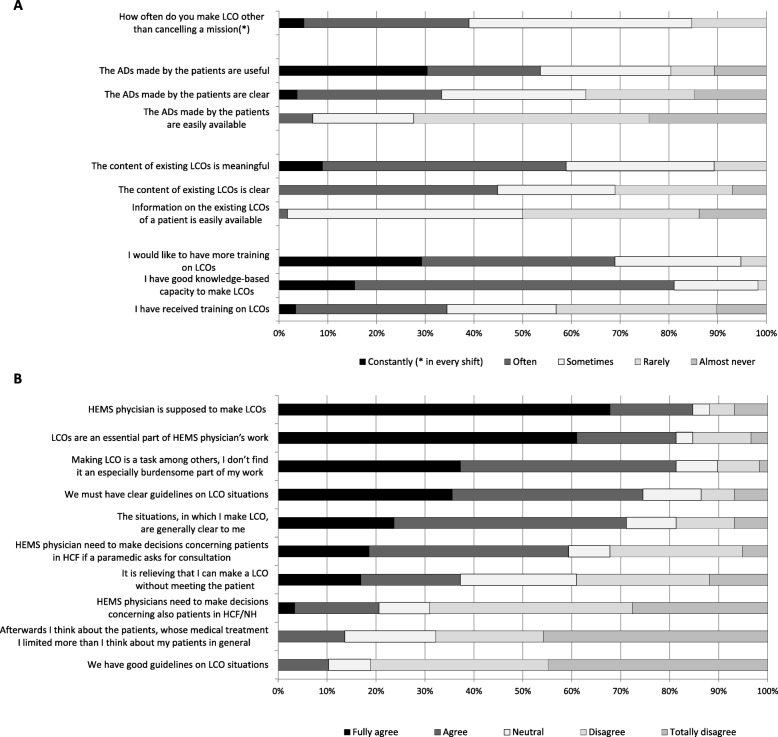
The opinions and experiences of Finnish HEMS physicians (*n* = 59) on prehospital limitations of care orders (LCOs). **a** presents how often they encounter some phenomena in their work and **b** presents how much the physicians agreed with certain claims

**Fig. 3:**
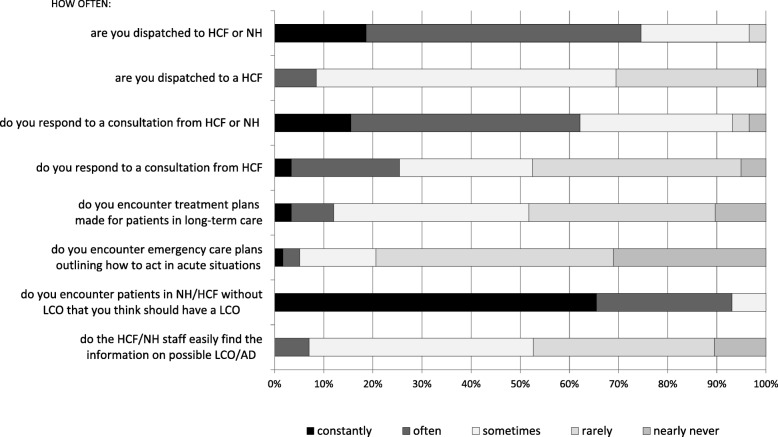
The opinions and experiences of Finnish HEMS physicians (*n* = 59) on missions designated to treat a patient in a healthcare facility (HCF) and nursing home (NH) and phone consultations from those locations made by a paramedic or HCF/NH staff. LCO is a limitation of care order made by the physician and AD is an advance directive made by the patient

There was some variation in the physicians’ opinions and experiences concerning LCOs. General LCO practices are presented in Fig. 1, the opinions and experiences on prehospital LCOs in Fig. 2 and the results concerning patients in HCFs and NHs in Fig. 3. The physicians perceived that their LCO was valid until the next physician’s evaluation, *n =* 31 (53%), during the adjacent hospitalization period, *n* = 13 (22%), only in the current situation, *n* = 9 (15%), and permanently, *n* = 2 (3%), while *n* = 1 (2%) selected ‘other’ and *n* = 3 (5%) did not reply.

Every other physician (*n =* 28, 49%) had sometimes decided not to do a medically justifiable LCO because they wanted to avoid a conflict with the patient, the next of kin, or HCF/NH staff. Two physicians (4%) answered that in this kind of situation they never make LCOs, but in contrast, 17 (30%) stated that they always make the necessary LCOs regardless of the possible conflict. Twelve (21%) physicians stated that they had never encountered that kind of situation and two (3%) did not respond to the question.

### Similarities between HEMS physicians’ attitudes, opinions, and experiences

We recognized only a few patterns in attitudes, opinions, and experiences between the physicians when we analysed the groups based on gender, age, and work experience. The bases did not differ in terms of the age or experience of the respondents. Although the portion of women varied between 14 and 56% within the bases, the gender distribution was generally similar (*p* = 0.363). The answers of female and male physicians differed to only one question. The women found that making LCOs is a task among others and not an especially burdensome part of work, as 60% of the women fully agreed with this claim and 30% agreed with the claim versus 26 and 51% of men (*p* = 0.024, Fisher). The total correlations between the physicians’ answers and age or work experience as physicians are shown in Table 2. The physicians with 20 years or more of work experience had fewer neutral answers compared to other physicians (see Additional file 2).

**Table 2:** The significant Spearman correlations between the Likert-scale questions or claims and the work experience or the age of HEMS physicians

	Correlation coefficient	*p*
The experience as physician in years in total		
Have a phone conversation with the next of kin in LCO situation	0.29	0.026
We have good guidance on LCO situations	−0.311	0.017
The situations, in which I make a LCO, are generally clear to me	−0.276	0.034
I have encountered emergency care plans made for patients in long-term care	0.269	0.041
I would like to have more education on LCOs	0.281	0.032
Age of physician in years		
Respond to a (phone)consultation from HCF or NH	0.349	0.010
The advance directives made by the patients are useful	−0.336	0.016

In the Likert-scale, “1” was fully agree/constantly, “3” is neutral/sometimes and “5” is totally disagree/almost never. If the correlation coefficient is positive, the more experienced physicians more often disagreed with the claim (more often chose the option number “5”) than less experienced physicians. If the correlation coefficient is negative, the more experienced physicians more often agreed with the claim (chose the option number “1”). LCO is a limitation of care order, HCF is a health care facility and NH is a nursing home

### Qualitative data

The majority of the HEMS physicians (*n* = 50, 85%) reported challenging patient groups or situations for LCOs that are shown in Table 3. An example of such a case is an acutely ill child with an intellectual disability and severe chronic comorbidities but no emergency care plan or LCO. The prominent aspect of prehospital LCO situations was that there is only a limited amount of information available when making LCOs in the field, and yet the features of LCO situations are variable (Table 3). Many physicians (*n* = 32, 54%) found it more difficult to make LCOs via telephone and not meeting the patient, a few (*n* = 2, 3%) found those situations easier, and for some (*n* = 6, 10%) there was no difference.

**Table 3:** Features of prehospital limitation of care order decisions

Features of prehospital limitation of care order decisions	Physicians who mentioned	
	*n*	(%)
Patient characteristics		
Challenging situations to make a LCO by patients’ characteristics	41	69
Children and adolescent	26	44
Severe comorbidities	16	27
-Malignancy	5	8
Disabled patients (incl. intellectual and developmental disabilities)	11	19
In nursing home or in health care facility	11	19
Aged	10	17
Decreased cognitive status	4	7
Existing DNAR without other LCO	2	3
Event characteristics		
Limited data in use in the situation	31	53
Importance of solving the baseline functional status	17	29
Acute situations (cardiac arrest, injury, drowning etc.)	17	29
Interaction/communication with the next-of-kin	15	25
Lacking treatment plans on patients in HCF/NH	5	8
The limited resources of EMS system and health care	4	7
Limited time in use in the situation	3	5

The first part shows patients for whom making a prehospital limitation of care orders (LCO) are the most challenging. Other features of prehospital LCO situations in general are presented in the lower part of the table. Repeating reduced expressions (=codes) were identified in the HEMS physicians’ qualitative answers and quantified. The numbers are physicians who mentioned a certain code, regardless of how many times that physician mentioned the code. DNAR is a ‘do not attempt cardiopulmonary resuscitation’ order, EMS is Emergency Medical Services, HCF is health care facility and NH is nursing home

Although the questionnaire did not demand that the respondents define LCO, many physicians did describe LCO decision-making. Twenty-two (37%) physicians expressed their personal principles or practices regarding how they make LCOs, seven (12%) wrote some definition for the term LCO, and 14 (24%) expressed an opinion on LCOs or how they should be made. The most controversial topic was whether or not a HEMS physician’s decision to cancel the HEMS mission could be considered as an LCO when reviewing the definitions of LCO. The physicians said they usually make LCOs concerning only life-sustaining therapies and some feel uncomfortable issuing other LCOs, such as ‘no transportation’. The physicians wished to have further education on the general guidelines and clear criteria for LCOs (*n* = 20, 34%) and training on legal issues (*n* = 12, 20%). Forty-three (73%) physicians suggested more education for HCF and/or NH staff on LCOs and end-of-life care issues. Only 16 (27%) suggested that paramedics should receive more education on LCOs.

## Discussion

This is the first multicentre study on HEMS physicians’ opinions, attitudes, and experiences regarding LCOs [11]. We had a special interest in situations where a HEMS physician encountered LCO decision-making concerning patients in HCFs and NHs. The main finding of the study was that though all the respondents make LCOs, the principles for LCOs in the prehospital setting are not clear, and opinions and practices differ between physicians. Every other physician had sometimes decided not to do a medically justifiable LCO because they wanted to avoid a possible conflict with the patient, next of kin or HCF/NH staff. The HEMS physicians perceived their LCOs to concern usually only life-sustaining atherapies, such as intensive care and cardiopulmonary resuscitation. Almost all (93%) physicians working in HEMS units often encounter patients in HCFs and NHs who do not have appropriate LCOs, at least from the perspective of the HEMS physicians.

### The experiences of HEMS physicians

Altering LCO definitions, various personal practices, as well as different opinions and experiences could not be fully explained by the respondents’ professional or sociodemographic backgrounds. The less experienced physicians more often found the existing guidance on LCO situations to be insufficient, called the next of kin when making LCOs, and had encountered emergency care plans made for patients in 24-h care. Younger physicians felt they answered phone consultations concerning patients from HCFs or NHs more often than older physicians. The experienced physicians had greater confidence to make LCOs and stronger opinions on topics related to LCOs that probably stem from their repeated exposure to LCO decision-making situations during their career. Yet it seems that the variation in attitudes mainly reflected the differences between individual physicians, which was also seen in another recent study [13]. The different working cultures between the bases may also regulate the individual’s decisions, especially in ethically or cognitively challenging situations involving prehospital LCO decisions [7, 30].

Interestingly, the baseline functional status was the most frequent patient-related reason for the provision of a LCO in this study. Defining a patient’s functional status and then adjusting the goals of care and estimating the prognosis of chronic illnesses may be challenging in a prehospital setting. Patients tend to evaluate their quality of life better than their physicians, and if the physician estimates the quality of life as poor, they are more inclined to withhold life-sustaining therapies [5]. Many physicians wished for more guidance on LCOs, which is understandable based on these results.

### The challenges of prehospital LCOs

Every other HEMS physician had at occasion decided not to limit the patients’ treatment in order to avoid a possible conflict. This phenomenon isn’t unique [31], but the solution to withhold LCO might not reflect the patient’s best interest. In addition to avoiding conflict, refraining from making an LCO might be due to preferentially avoiding prolonged scene times, but in this study the physicians very seldom described the lack of time affecting their decision-making. Instead, the physicians reported that information regarding the medical history of the patient in the prehospital setting is minute and scattered, which usually leads to full treatment and transportation to a hospital rather than to hasty LCOs as the early withdrawal of life-sustaining therapies may lead to excessive mortality [32]. The physicians have access to the patient records while working on the helicopter bases, but their access to any patient records in the field is limited due to the absence of mobile patient records.

### HEMS missions to HCFs and NHs

The challenging nature of LCOs in HCFs and NHs may be the reason why HEMS physicians are sometimes asked to make the end-of-life decisions, though it should be the responsibility of the patient’s treating physician. Discussion on end-of-life topics is difficult and LCOs may have a negative impact on the patient [5, 7, 33]. In addition, physicians at HCFs and NHs may overestimate the prognoses of their patients [34] and yet may not be as familiar with treating acutely ill patients as HEMS physicians are. In addition to either offering or limiting life-sustaining therapies while on the scene, HEMS physicians also provide their competence in clinical decision-making when evaluating and treating severely ill patients [7, 35].

The clinical relevance of HEMS physicians treating patients in HCFs and NHs is significant as 75% of physicians answered that they are often dispatched to treat patients in HCFs and NHs. This patient population is remarkable, and as the Finnish population ages, the number of people in HCFs and NHs will remain high. Among people over 75 years old, 50,373 (9%) lived in 24-h care in Finland as of 31st Dec 2016 [36]. The biggest client group in 24-h care consists of aged patients with modern to severe dementia who often have simultaneous comorbidities [24]. Their survival from critical illness is low, but they often don’t have appropriate emergency care plans for acute situations, LCOs, or sufficient palliation [3, 5, 34]. Finnish people aged 70 years or older usually die in a HCF, typically in a municipal health center in-patient ward, and 70 to 80% of aged people are transferred to a HCF during the last 3 months of their life [21, 24]. Nevertheless, in NHs, EMS providers are often needed to provide palliation and to ease the distress of the HN staff or to execute those transfers at the end of life [15]. Therefore, the HEMS physicians’ perception of deficient treatments plans, end-of-life care plans, and emergency care plans is understandable [26]. Unfortunately, the low prevalence of these plans seems to reflect the status of end-of-life care quality, equality, and availability [3, 24]. This may lead to excess suffering and healthcare costs, and increases the risk of concurrent EMS missions [10, 37].

### Strengths and limitations

The major strength of this study was that almost all Finnish HEMS physicians participated in the study. As the exact definition of LCO and the content of different LCOs are unclear, giving any definitions for this study would have constituted an intervention, and we wanted to find all possible heterogeneity in the answers. This study was conducted among Finnish HEMS physicians. Although the professional background of the respondents was fairly similar to that of other European HEMS physicians, this sets the frames for the overall generalizability of these studies; results may not apply in countries with different clinical practices or arrangements of healthcare, EMS systems, and care of the aged [1, 2, 13]. Based on our results, more data on other countries are urgently warranted.

## Conclusions

Making LCOs is an important but often invisible part of HEMS physicians’ work in Finland. These physicians often treat patients in NHs and HCFs, and they stated that among those patients, emergency care plans and LCOs should have been made in advance more often than occur at the moment. The physicians want to avoid conflicts and are reluctant to limit treatments in indistinct circumstances. There is variation in LCO practices and attitudes based partly on the experience of the physicians, but the differences are mostly caused by the varying individual working procedures and deficient guidelines. Further research is needed to determine the true frequency and content of prehospital LCOs.

## Additional files

Additional file 1: The study survey with English translations. (DOCX 50 kb)

Additional file 2: The differences in opinions and practices between the most experienced quartile of Finnish HEMS physicians (with 20 years or more of work experience as physician in total, *n* = 12) and other physicians (*n* = 47) analysed with Fisher’s exact test. (DOCX 16 kb)

## Data Availability

Please contact the author for data requests.

## Abbreviations

AD: Advance directive
ALS: Advanced life support
CPR: Cardiopulmonary resuscitation
DNAR: Do-not-attempt-resuscitation
EMS: Emergency medical services
HCF: Health care facility
HEMS: Helicopter emergency medical services
IQR: Interquartile range
LCO: Limitation of care order
NH: Nursing home
