# Supplementary material for: Limitation of treatment in prehospital care – the experiences of helicopter emergency medical service physicians in a nationwide multicentre survey
Source: Scand J Trauma Resusc Emerg Med. 2019 Oct 2;27:89. doi: 10.1186/s13049-019-0663-x (PMC6775669; doi:10.1186/s13049-019-0663-x)
Supplement: Supplementary file 1 — The study survey with English translations. (DOCX 50 kb) [file 13049_2019_663_MOESM1_ESM.docx]

**Additional File 1.** The English translation of the questionnaire.

| Question number | Question + answers | | | |  |
| --- | --- | --- | --- | --- | --- |
| 1 | Saat tehtävän hoitolaitokseen | | | |  |
|  | How often are you dispatched to any nursing home (NH) or healthcare facility (HCF) | | | |  |
|  |  | In every shift | | |  |
|  |  | often | | |  |
|  |  | Sometimes | | |  |
|  |  | Rarely | | |  |
|  |  | Almost never | | |  |
| 2 | Saat tehtävän sairaalaan tai terveyskeskukseen | | | |  |
|  | How often are you dispatched to a hospital or other HCF | | | |  |
|  |  | In every shift | | |  |
|  |  | often | | |  |
|  |  | Sometimes | | |  |
|  |  | Seldom | | |  |
|  |  | Almost never | | |  |
| 3 | Vastaat ensihoitajan tai laitoksen henkilökunnan konsultaatioon hoitolaitoksesta | | | | |
|  | How often do you respond to a (phone)consultation from HCF or NH made by a paramedic or NH/HCF staff | | | | |
|  |  | In every shift | | |  |
|  |  | often | | |  |
|  |  | Sometimes | | |  |
|  |  | Seldom | | |  |
|  |  | Almost never | | |  |
| 4 | Vastaat ensihoitajan tai laitoksen henkilökunnan konsultaatioon sairaalasta tai terveyskeskuksesta | | | | |
|  | How often do you respond to a (phone)consultation from HCF made by a paramedic or HCF staff | | | | |
|  |  | In every shift | | |  |
|  |  | often | | |  |
|  |  | Sometimes | | |  |
|  |  | Seldom | | |  |
|  |  | Almost never | | |  |
| 5 | Teet muun hoidonrajauksen kuin peruutuksen millä tahansa tehtävällä | | | |  |
|  | How often do you make limitation of care orders (LCOs) other than cancelling a mission | | | |  |
|  |  | In every shift | | |  |
|  |  | often | | |  |
|  |  | Sometimes | | |  |
|  |  | Seldom | | |  |
|  |  | Almost never | | |  |
| 6 | Mitkä ovat hoidonrajausten ja peruutusten taustalla olevat potilaaseen liittyvät seikat? Valitse kuhunkin väittämään yksi parhaiten sopiva vaihtoehto. | | | | |
|  | What are the major patient-related reasons for LCOs and cancellations on emergency missions? Please choose the best suitable option for each statement. | | | | |
| 6.a | Potilaan ikä | | | |  |
|  | Age of the patient | | | |  |
|  |  | Constantly | | |  |
|  |  | often | | |  |
|  |  | Sometimes | | |  |
|  |  | Rarely | | |  |
|  |  | Never | | |  |
| 6.b | Potilaan yleiskunto tai toimintakyky | | | |  |
|  | The baseline functional status of the patient | | | |  |
|  |  | Constantly | | |  |
|  |  | often | | |  |
|  |  | Sometimes | | |  |
|  |  | Rarely | | |  |
|  |  | Never | | |  |
| 6.c | Vaikeat tai lukuisat perussairaudet | | | |  |
|  | Multiple or severe comorbidities | | | |  |
|  |  | Constantly |  | |  |
|  |  | often |  | |  |
|  |  | Sometimes |  | |  |
|  |  | Rarely |  | |  |
|  |  | Never |  | |  |
| 6.d | Potilas on hoivakodissa tai hoitolaitoksessa | |  | |  |
|  | The patient is in a HCF or NH | |  | |  |
|  |  | Constantly |  | |  |
|  |  | often |  | |  |
|  |  | Sometimes |  | |  |
|  |  | Rarely |  | |  |
|  |  | Never |  | |  |
| 6.e | Kokonaistilanteen toivottomuus | |  | |  |
|  | Futility of the overall situation | |  | |  |
|  |  | Constantly |  | |  |
|  |  | often |  | |  |
|  |  | Sometimes |  | |  |
|  |  | Rarely |  | |  |
|  |  | Never |  | |  |
| 6.f | Potilas ei ole päivystyksellisen hoidon tarpeessa | |  | |  |
|  | The patient is not in need for emergency medical care | |  | |  |
|  |  | Constantly |  | |  |
|  |  | often |  | |  |
|  |  | Sometimes |  | |  |
|  |  | Rarely |  | |  |
|  |  | Never |  | |  |
| 7 | Mitkä ovat hoidonrajausten ja peruutusten taustalla olevat järjestelmään (operatiivinen tilanne, riskinarvio tms.) liittyvät seikat? Valitse kuhunkin väittämään yksi parhaiten sopiva vaihtoehto. | | | |  |
|  | What are the major system-related reasons for LCOs and cancellations on emergency mission (operative situation, risk assessment and so on)? Please choose the best suitable option for each statement. | | | |  |
| 7.a | Ongelma ei ole lääketieteellinen | |  | |  |
|  | The problem is not medical | |  | |  |
|  |  | Constantly |  | |  |
|  |  | often |  | |  |
|  |  | Sometimes |  | |  |
|  |  | Rarely |  | |  |
|  |  | Never |  | |  |
| 7.b | Ensihoito hälytettiin kohteeseen turhaan | |  | |  |
|  | EMS was dispatched to the scene unnecessarily | |  | |  |
|  |  | Constantly |  | |  |
|  |  | often |  | |  |
|  |  | Sometimes |  | |  |
|  |  | Rarely |  | |  |
|  |  | Never |  | |  |
| 7.c | Potilaalla on mahdollisuus saada hoitoa muuten kuin ensihoitojärjestelmän kautta | | | |  |
|  | The patient can receive (medical) treatment different way than via EMS | | | |  |
|  |  | Constantly |  | |  |
|  |  | often |  | |  |
|  |  | Sometimes |  | |  |
|  |  | Rarely |  | |  |
|  |  | Never |  | |  |
| 7.d | HEMS yksikköä ei tarvita kohteessa | |  | |  |
|  | HEMS unit is not needed on scene | |  | |  |
|  |  | Constantly |  | |  |
|  |  | often |  | |  |
|  |  | Sometimes |  | |  |
|  |  | Rarely |  | |  |
|  |  | Never |  | |  |
| 7.e | Resurssit (aika, yksiköt) eivät riitä hoitamaan potilasta | |  | |  |
|  | Resources (time, units) are too limited to treat the patient | |  | |  |
|  |  | Constantly |  | |  |
|  |  | often |  | |  |
|  |  | Sometimes |  | |  |
|  |  | Rarely |  | |  |
|  |  | Never |  | |  |
| 7.f | Potilas on liian kaukana | |  | |  |
|  | The distance to the location of patient is too long | |  | |  |
|  |  | Constantly |  | |  |
|  |  | often |  | |  |
|  |  | Sometimes |  | |  |
|  |  | Rarely |  | |  |
|  |  | Never |  | |  |
| 8 | Oletko hoidonrajaustilanteissa yhteydessä omaisiin? | |  | |  |
|  | Do you communicate with the next-of-kin in LCO situations? | |  | |  |
| 8.a | Keskustelen kasvotusten | |  | |  |
|  | I’ll have a face-to-face conversation | |  | |  |
|  |  | Constantly |  | |  |
|  |  | often |  | |  |
|  |  | Sometimes |  | |  |
|  |  | Rarely |  | |  |
|  |  | Never |  | |  |
| 8.b | Keskustelen puhelimessa | |  | |  |
|  | I’ll have a phone conversation | |  | |  |
|  |  | Constantly |  | |  |
|  |  | often |  | |  |
|  |  | Sometimes |  | |  |
|  |  | Rarely |  | |  |
|  |  | Never |  | |  |
| 9 | Olen kohdannut erimielisyyttä hoidonrajaamisessa? | |  | |  |
|  | Have you confronted dissonance in making LCOs? | |  | |  |
| 9.a | Potilaan kanssa | |  | |  |
|  | With the patient | |  | |  |
|  |  | Constantly |  | |  |
|  |  | often |  | |  |
|  |  | Sometimes |  | |  |
|  |  | Rarely |  | |  |
|  |  | Never |  | |  |
| 9.b | Omaisten kanssa | |  | |  |
|  | With the next of kin | |  | |  |
|  |  | Constantly |  | |  |
|  |  | often |  | |  |
|  |  | Sometimes |  | |  |
|  |  | Rarely |  | |  |
|  |  | Never |  | |  |
| 9.c | Ensihoitajien kanssa | |  | |  |
|  | With paramedics | |  | |  |
|  |  | Constantly |  | |  |
|  |  | often |  | |  |
|  |  | Sometimes |  | |  |
|  |  | Rarely |  | |  |
|  |  | Never |  | |  |
| 9.d | Toisten ensihoitolääkärien kanssa esim. vuoronvaihdossa | |  | |  |
|  | With other EMS physicians when for example changing shifts | |  | |  |
|  |  | Constantly |  | |  |
|  |  | often |  | |  |
|  |  | Sometimes |  | |  |
|  |  | Rarely |  | |  |
|  |  | Never |  | |  |
| 9.e | Hoitolaitoksessa henkilökunnan kanssa | |  | |  |
|  | With HCF or NH staff | |  | |  |
|  |  | Constantly |  | |  |
|  |  | often |  | |  |
|  |  | Sometimes |  | |  |
|  |  | Rarely |  | |  |
|  |  | Never |  | |  |
| 9.f | Vastaanottavan hoitolaitoksen kanssa | |  | |  |
|  | With the HCF where a patient is transferred to | |  | |  |
|  |  | Constantly |  | |  |
|  |  | often |  | |  |
|  |  | Sometimes |  | |  |
|  |  | Rarely |  | |  |
|  |  | Never |  | |  |
| 9.g | VALVIRA:n tms. valvovan viranomaisen kanssa | |  | |  |
|  | With the National Supervising Authority for Welfare and Health or other authorities | |  | |  |
|  |  | Constantly |  | |  |
|  |  | often |  | |  |
|  |  | Sometimes |  | |  |
|  |  | Rarely |  | |  |
|  |  | Never |  | |  |
| 10 | Miten usein jätät jonkun lääketieteellisesti perustellun hoidonrajauksen tekemättä, koska kohtaat erimielisyyttä potilaan/omaisen/hoitohenkilökunnan kanssa ja haluat välttää konfliktin tai jatkoseuraamukset? (avoin kysymys) | | | |  |
|  | How often do you choose not to make a LCO that would be medically justifiable, because you have discrepancy with the patient/next of kin/personnel and you want to avoid conflict or further consequence? (open question) | | | |  |
| 11 | Ensihoitolääkärin tekemä hoidonrajaus pysyy voimassa | |  | |  |
|  | The LCO made by HEMS physician is valid | |  | |  |
|  |  | Only in this situation |  | |  |
|  |  | Until the next physician's evaluation |  | |  |
|  |  | During the adjacent hospitalization |  | |  |
|  |  | Permanently |  | |  |
|  |  | Other, what? |  | |  |
| 12 | Hoidonrajaukset ovat olennainen osa ensihoitolääkärin työtä. | |  | |  |
|  | LCOs are an essential part of HEMS physician’s work | |  | |  |
|  |  | Fully agree |  | |  |
|  |  | Somewhat agree |  | |  |
|  |  | Neutral |  | |  |
|  |  | somewhat disagree |  | |  |
|  |  | Totally disagree |  | |  |
| 13 | Ensihoitolääkärin kuuluu tehdä hoidonrajauksia | |  | |  |
|  | HEMS phycisian is supposed to make LCOs | |  | |  |
|  |  | Fully agree |  | |  |
|  |  | Somewhat agree |  | |  |
|  |  | Neutral |  | |  |
|  |  | somewhat disagree |  | |  |
|  |  | Totally disagree |  | |  |
| 14 | On helpottavaa, että voin rajata hoitoa myös potilasta tapaamatta | |  | |  |
|  | It is relieving that I can make a LCO without meeting the patient | |  | |  |
|  |  | Fully agree |  | |  |
|  |  | Somewhat agree |  | |  |
|  |  | Neutral |  | |  |
|  |  | somewhat disagree |  | |  |
|  |  | Totally disagree |  | |  |
| 15 | Meillä on hyvät ohjeistukset hoidonrajaustilanteisiin | |  | |  |
|  | We have good guidelines on LCO situations | |  | |  |
|  |  | Fully agree |  | |  |
|  |  | Somewhat agree |  | |  |
|  |  | Neutral |  | |  |
|  |  | somewhat disagree |  | |  |
|  |  | Totally disagree |  | |  |
| 16 | Meillä pitää olla selkeät ohjeistukset hoidonrajaustilanteisiin | | | |  |
|  | We must have clear guidelines on LCO situations | | | |  |
|  |  | Fully agree |  | |  |
|  |  | Somewhat agree |  | |  |
|  |  | Neutral |  | |  |
|  |  | somewhat disagree |  | |  |
|  |  | Totally disagree |  | |  |
| 17 | Ensihoitolääkäreiden tulee ottaa kantaa myös erilaisissa hoitolaitoksissa oleviin potilaisiin | | | |  |
|  | HEMS physicians need to make decisions concerning also patients in different kinds of HCFs and NHs | | | |  |
|  |  | Fully agree |  | |  |
|  |  | Somewhat agree |  | |  |
|  |  | Neutral |  | |  |
|  |  | somewhat disagree |  | |  |
|  |  | Totally disagree |  | |  |
| 18 | Ensihoitolääkäreiden tulee ottaa kantaa sairaaloissa ja terveyskeskuksissa olevien potilaiden hoitoon, mikäli ensihoitaja konsultoi | | | |  |
|  | HEMS physician need to make decisions concerning patients in HCF if a paramedic asks for a consultation | | | |  |
|  |  | Fully agree |  | |  |
|  |  | Somewhat agree |  | |  |
|  |  | Neutral |  | |  |
|  |  | somewhat disagree |  | |  |
|  |  | Totally disagree |  | |  |
| 19 | Hoidon rajaaminen on minulle työtehtävä muiden joukossa, en koe sitä erityisen raskaana osana työtäni | | | |  |
|  | Making LCO is a task among others, I don’t find it an especially burdensome part of my work | | | |  |
|  |  | Fully agree |  | |  |
|  |  | Somewhat agree |  | |  |
|  |  | Neutral |  | |  |
|  |  | somewhat disagree |  | |  |
|  |  | Totally disagree |  | |  |
| 20 | Tilanteet, joissa hoitoa rajaan, ovat minulle yleensä varsin selkeitä | | | |  |
|  | The situations, in which I make LCOs, are generally clear to me | | | |  |
|  |  | Fully agree |  | |  |
|  |  | Somewhat agree |  | |  |
|  |  | Neutral |  | |  |
|  |  | somewhat disagree |  | |  |
|  |  | Totally disagree |  | |  |
| 21 | Mietin jälkikäteen potilaita, joiden hoitoa rajasin, enemmän kuin mietin hoitamiani potilaita yleensä | | | |  |
|  | Afterwards I think about the patients, whose medical treatment I limited more than I think about my patients in general | | | |  |
|  |  | Fully agree |  | |  |
|  |  | Somewhat agree |  | |  |
|  |  | Neutral |  | |  |
|  |  | somewhat disagree |  | |  |
|  |  | Totally disagree |  | |  |
| 22 | Onko joku tilanne tai potilasryhmä, jossa hoidonrajausten teko on erityisen haastavaa? (avoin kysymys) | | | |  |
|  | Is there a situation or patient group where you find making of LCO especially challenging? (open question) | | | |  |
| 23 | Verrattuna edelliseen kysymykseen, miten tilanteet ja syyt eroavat, jos joudut rajaamaan hoitoa potilasta tapaamatta? (avoin kysymys) | | | |  |
|  | Compared to the previous question, how do situations and reasons differ if you have to make a LCO without meeting the patient? (open question) | | | |  |
| 24 | Tieto potilaalle tehdyistä rajauksista on helposti saatavilla | |  | |  |
|  | Information on the existing LCO of a patient is easily available | |  | |  |
|  |  | Constantly |  | |  |
|  |  | Often |  | |  |
|  |  | Sometimes |  | |  |
|  |  | Seldom |  | |  |
|  |  | Almost never |  | |  |
| 25 | Hoidonrajausten sisältö on selkeä | |  | |  |
|  | The content of LCO is clear | |  | |  |
|  |  | Constantly |  | |  |
|  |  | Often |  | |  |
|  |  | Sometimes |  | |  |
|  |  | Seldom |  | |  |
|  |  | Almost never |  | |  |
| 26 | Hoidonrajausten sisältö on mielekäs | |  | |  |
|  | The content of LCOs is meaningful | |  | |  |
|  |  | Constantly |  | |  |
|  |  | Often |  | |  |
|  |  | Sometimes |  | |  |
|  |  | Seldom |  | |  |
|  |  | Almost never |  | |  |
| 27 | Potilaiden laatimat hoitotahdot ovat helposti saatavilla | |  | |  |
|  | The advance directives made by the patients are easily available | |  | |  |
|  |  | Constantly |  | |  |
|  |  | Often |  | |  |
|  |  | Sometimes |  | |  |
|  |  | Seldom |  | |  |
|  |  | Almost never |  | |  |
| 28 | Potilaiden laatimat hoitotahdot ovat selkeitä | |  | |  |
|  | The advance directives made by the patients are clear | |  | |  |
|  |  | Constantly |  | |  |
|  |  | Often |  | |  |
|  |  | Sometimes |  | |  |
|  |  | Seldom |  | |  |
|  |  | Almost never |  | |  |
| 29 | Potilaiden laatimista hoitotahdoista on hyötyä | |  | |  |
|  | The advance directives made by the patients are useful | |  | |  |
|  |  | Constantly |  | |  |
|  |  | Often |  | |  |
|  |  | Sometimes |  | |  |
|  |  | Seldom |  | |  |
|  |  | Almost never |  | |  |
| 30 | Olen kohdannut pitkäaikaishoitopotilaalle laadittuja hoitosuunnitelmia | | | |  |
|  | I have encountered treatment plans made for patients in long-term care | | | |  |
|  |  | Constantly |  | |  |
|  |  | Often |  | |  |
|  |  | Sometimes |  | |  |
|  |  | Seldom |  | |  |
|  |  | Almost never |  | |  |
| 31 | Olen kohdannut em. hoitosuunnitelmia, joissa otetaan kantaa toimintaan akuuteissa tilanteissa | | | |  |
|  | I have encountered treatment plans outlining how to act in emergency situations | | | |  |
|  |  | Constantly |  | |  |
|  |  | Often |  | |  |
|  |  | Sometimes |  | |  |
|  |  | Seldom |  | |  |
|  |  | Almost never |  | |  |
| 32 | Kohtaan hoitolaitoksessa olevia potilaita, joilla ei ole mitään hoidonrajauksia, vaikka mielestäni olisi pitänyt olla | | | |  |
|  | How often do you encounter patients in NH/HCF without LCO that you think should have a LCO | | | |  |
|  |  | Constantly |  |  |  |
|  |  | Often |  | |  |
|  |  | Sometimes |  | |  |
|  |  | Seldom |  | |  |
|  |  | Almost never |  | |  |
| 33 | Hoitohenkilökunta löytää helposti tiedot hoitotahdosta tai potilaalle mahdollisesti tehdyistä hoidonrajauksista | | | |  |
|  | The HCF/NH staff easily find the information on possible LCO or advance directives of the patient | | | |  |
|  |  | Constantly |  | |  |
|  |  | Often |  | |  |
|  |  | Sometimes |  | |  |
|  |  | Seldom |  | |  |
|  |  | Almost never |  | |  |
| 34 | Olen saanut koulutusta hoidonrajauksista | |  | |  |
|  | I have received education on LCOs | |  | |  |
|  |  | Fully agree |  | |  |
|  |  | Somewhat agree |  | |  |
|  |  | Neutral |  | |  |
|  |  | somewhat disagree |  | |  |
|  |  | Totally disagree |  | |  |
| 35 | Minulla on hyvät tiedolliset valmiudet tehdä hoidonrajauksia | | | |  |
|  | I have good knowledge-based capacity to make LCOs | | | |  |
|  |  | Fully agree |  | |  |
|  |  | Somewhat agree |  | |  |
|  |  | Neutral |  | |  |
|  |  | somewhat disagree |  | |  |
|  |  | Totally disagree |  | |  |
| 36 | Haluaisin lisää koulutusta hoidonrajauksista | |  | |  |
|  | I would like to have more education on LCOs | |  | |  |
|  |  | Fully agree |  | |  |
|  |  | Somewhat agree |  | |  |
|  |  | Neutral |  | |  |
|  |  | somewhat disagree |  | |  |
|  |  | Totally disagree |  | |  |
| 37 | Millaisista asioista haluaisit tietoa? (avoin kysymys) | | | |  |
|  | What kind of topics would you like to have more information on? (open question) | | | |  |
| 38 | Kenelle tai millaisille toimijoille toivoisit koulutusta annettavan? Mistä aiheesta? (avoin kysymys) | | | |  |
|  | Who or what kind of actors would you wish to receive the education? About what topics? (open question) | | | |  |
