# Supplementary material for: Limitation of treatment in prehospital care – the experiences of helicopter emergency medical service physicians in a nationwide multicentre survey
Source: Scand J Trauma Resusc Emerg Med. 2019 Oct 2;27:89. doi: 10.1186/s13049-019-0663-x (PMC6775669; doi:10.1186/s13049-019-0663-x)
Supplement: Supplementary file 2 — The differences in opinions and practices between the most experienced quartile of Finnish HEMS physicians (with 20 years or more of work experience as physician in total, n = 12) and other physicians (n = 47) analysed with Fisher’s exact test. (DOCX 16 kb) [file 13049_2019_663_MOESM2_ESM.docx]

**Additional File 2.** The differences in opinions and practices between the most experienced quartile of Finnish HEMS physicians (with 20 years or more work experience as physician in total, *n*=12) and other physicians (*n*=47) analysed with Fisher’s exact test.

| The question or claim with significant difference | The distribution of answers on five-point Likert-scale (%) | | | | | | | |
| --- | --- | --- | --- | --- | --- | --- | --- | --- |
|  | Fully agree/constantly | |  | Disagree/never | | | | |
|  | 1 | | 2 | 3 | 4 | 5 | | p |
| Reasons for LCOs and cancellations on HEMS missions: |  |  |  |  |  |  |  | |
| Age of the patient | Most experienced | 17 | 17 | 17 | 25 | 25 | 0.002 | |
|  | Less experienced | 2 | 49 | 38 | 9 | 2 |  |  |
| The patient is in an HCF or NH | Most experienced | 0 | 75 | 0 | 17 | 8 | 0.003 | |
|  | Less experienced | 13 | 62 | 26 | 0 | 0 |  |  |
| Multiple or severe comorbidities | Most experienced | 42 | 17 | 42 | 0 | 0 | 0.004 | |
|  | Less experienced | 57 | 38 | 4 | 0 | 0 |  |  |
| The patient is not in need for emergency medical care | Most experienced | 0 | 67 | 17 | 8 | 8 | 0.003 | |
|  | Less experienced | 9 | 14 | 47 | 28 | 2 |  |  |
|  |  |  |  |  |  |  |  | |
| LCOs are an essential part of HEMS  physician’s work | Most experienced | 67 | 0 | 17 | 8 | 8 | 0.016 | |
|  | Less experienced | 60 | 26 | 0 | 13 | 2 |  |  |
| It is relieving that I can make a LCO without meeting the patient | Most experienced | 17 | 0 | 33 | 17 | 33 | 0.035 | |
|  | Less experienced | 17 | 26 | 21 | 30 | 6 |  |  |
| HEMS physician need to make decisions concerning patients in HCF if a paramedic asks for consultation | Most experienced | 42 | 8 | 8 | 33 | 8 | 0.031 | |
|  | Less experienced | 13 | 49 | 9 | 26 | 4 |  |  |
| The situations, in which I make a LCO, are generally clear to me | Most experienced | 50 | 33 | 0 | 0 | 17 | 0.033 | |
|  | Less experienced | 17 | 51 | 13 | 15 | 4 |  |  |
| I have good knowledge-based capacity to make LCOs | Most experienced | 33 | 42 | 17 | 8 | 0 | 0.035 | |
|  | Less experienced | 11 | 72 | 17 | 0 | 0 |  |  |

LCO is a limitation of care order, HCF is a health care facility and NH is a nursing home.
